# Prognostic for hydraulic pump based upon DCT-composite spectrum and the modified echo state network

**DOI:** 10.1186/s40064-016-2933-7

**Published:** 2016-08-08

**Authors:** Jian Sun, Hongru Li, Baohua Xu

**Affiliations:** Shijiazhuang Mechanical Engineering College, Shijiazhuang, 050003 People’s Republic of China

**Keywords:** Prognostic, Composite spectrum analysis, ESN, Small world networks

## Abstract

Prognostic is a key step of the condition-based maintenance (CBM). In order to improve the predicting performance, a novel method for prognostic for the hydraulic pump is proposed in this paper. Based on the improvement of the traditional composite spectrum, the DCT-composite spectrum (DCS) fusion algorithm is initially presented to make fusion of multi-channel vibration signals. The DCS composite spectrum entropy is extracted as the feature. Furthermore, the modified echo state networks (ESN) model is established for prognostic using the extracted feature. The reservoir is updated and the elements of the neighboring matrix are redefined for improving predicting accuracy. Analysis of the application in the hydraulic pump degradation experiment demonstrates that the proposed algorithm is feasible and is meaningful for CBM.

## Background

Conditional-based maintenance (CBM) is one of the modern maintenance concepts. The open system architecture for CBM organization divides a standard CBM system into several various layers with technical modules (Ahmad and Kamaruddin 2012). The core functions, corresponding to maintenance decisions among the architecture, can be summarized as condition monitoring, health assessment and prognostics (Coraddu et al. 2015; Sun et al. 2015). With the fast development of industry and the international market, especially the areas of complex plants such as shipbuilding and aircraft, CBM recommends timely and accurate maintenance actions based on the information obtained from condition monitoring (Altosole et al. 2014). Therefore, more and more advanced machine learning approaches have been applied in CBM for better improve the reliability, such as support vector machine and information fusion algorithms (Coraddu et al. 2014; Niu et al. 2010; Widodo and Yang 2007). Furthermore, some real-time on-line diagnostics have also been employed to extract sensitive information of the current operational status for identify the detailed failures (Gulen et al. 2002). In order to meet the requirement of CBM for better improving the hydraulic system reliability, it is necessary to search for an effective method for accurate prognostics of the hydraulic pump.

Normal algorithms for prognostics mainly contains two categories (EI-Thalji and Jantunen 2015): the one are algorithms based on models, such as the physics model algorithm (Jin et al. 2013), the auto-regressive and moving average algorithm (ARMA) (Yu et al. 2013), and the auto-regressive integrated moving average algorithm (ARIMA) (Li and Hu 2012). Others are algorithms based on neural intelligence, such as the BP neural network (Shuran and Shujin 2011), the recurrent neural network (RNN) (Cao et al. 2012), the extreme learning machine (ELM) (Wang et al. 2011), and the echo state networks (ESN) (Park et al. 2014). The physics model algorithm is capable of making prediction of the operating trends and the possible failures. However, it requires the mathematic model to be established extremely accurately, which can hardly be fulfilled in engineering application. ARMA is one of the typical random time series predicting methods. Limited by its computing theory, ARMA performs effectively only for stationary series (Zhu et al. 2014). Being the improvement of ARMA, ARIMA can be applied in non-stationary series. However, it is lack of the ability to uncover the system inner mechanism (Liu et al. 2012). Compared with the algorithms based on models, BP neural network performs better non-linear fitting ability, whereas, the predicting for non-linear and time-varying series is limited (Ren et al. 2014). Considering time series correlation, the prognostics by RNN are better than the BP. Since the back propagation is still employed, RNN is easy to trap in local minimums (Wu and Zhu 2014). ELM is the high-speed learning algorithm with the one-layer feed-forward neural networks. Instead of the iteration strategy of gradient descent, ELM only requires setting the number of hidden layer nodes and the driving function. It has advantages such as simple parameters, fast learning and better searching ability. Influenced by the singular values decomposition in ELM, if the number of hidden layer nodes is too large, the computing complexity will obviously increase leading to the reduction of the learning efficiency. Additionally, the random selection of input weights vectors and hidden layer nodes’ thresholds may also affect the network stability and the prediction accuracy (Wang and Han 2014). ESN is a novel recurrent neural network algorithm proposed by Jaeger and Haas (2004). It applies the reservoir as the inner network to inspire complex nonlinear state space for increasing the nonlinear fitting ability. In the ESN training process, the connecting weights keep the same. The computing complexity is reduced and the issue of trapping into local minimums is avoided. Therefore, ESN can fundamentally meet the predicting requirements of hydraulic pumps with nonlinear and non-stationary vibration signals. Since neurons in the reservoir are randomly and sparsely connected, the guidance and purpose of the network are poor. To solve this problem, the small world networks (Friedkin 2011) are introduced to make modification on the structure of the ESN reservoir in this paper. Meanwhile, the elements in neighboring matrix are redefined based on distance and random factor to further improve the generalization ability.

The realization of prognostic lays foundation of the extraction of effective features. However, the fault information in one-channel vibration signal is incomplete. The effective way to obtain accurate failure information is to make fusion of multi-channel vibration signals for extracting appropriate features (Safizadeh and Latifi 2014). As a novel spectrum analysis algorithm proposed recently, composite spectrum (CS) is able to realize information fusion of various signals by calculating correlative index and mutual power spectrum of neighboring signals (Elbhbah and Sinha 2013). It can effectively restrain noises, and obtain sensitive feature information of signals simultaneously (Akilu et al. 2014). Considering the structural characters of hydraulic pumps, some modification is made on the CS algorithm in this paper to improve feature sensitivity.

Consequently, a novel method for prognostic for hydraulic pump based on discrete cosine transform—composite spectrum (DCS) and the modified ESN is proposed. Contributions of this article are summarized hereinafter. In “Feature extraction based on DCS fusion algorithm” section, the DCS fusion algorithm is presented and the method for the extracting DCS composite spectrum entropy is illustrated in detail; in “Fault predicting based on INW–ESN” section, we make modification on ESN and propose the INW–ESN model for failure prediction; in “Experimental validation” section, we confirm the results through the experiment of hydraulic pump; and in “Conclusions” section, we draw some conclusions.

## Feature extraction based on DCS fusion algorithm

### The proposed DCS fusion algorithm

Assuming that the number of sampled signals is *B*. Each signal is equally divided into $n_{s}$ segments, and then Fourier transform is carried out on each segment. The CS is defined as (Elbhbah and Sinha 2013):

1 $$S_{CS} (f_{k} ) = \sum\limits_{r = 1}^{{n_{s} }} {X_{CS}^{r} (f_{k} )X_{CS}^{r*} (f_{k} )} /n_{s}$$

where, $X_{CS}^{r} (f_{k} )$ and $X_{CS}^{r *} (f_{k} )$ respectively denote the CS Fourier transform coefficient and its complex conjugate for the *r*th segment of all *B* signals. $X_{CS}^{r} (f_{k} )$ can be achieved by Eq. (2).

2 $$X_{CS}^{r} (f_{k} ) = \sqrt {(S_{{x_{1} \gamma_{12} x_{2} }}^{r} (f_{k} )S_{{x_{2} \gamma_{23} x_{3} }}^{r} (f_{k} ) \cdots S_{{x_{B - 1} \gamma_{(B - 1)B} x_{B} }}^{r} (f_{k} ))^{1/(B - 1)} }$$

where, $\gamma_{12}^{2} ,\gamma_{23}^{2} , \ldots ,\gamma_{(B - 1)B}^{2}$ respectively represent the coherences between neighboring signals (signal1–signal2, signal2–signal3, …). $S_{{x_{1} \gamma_{12} x_{2} }}^{r} (f_{k} ),S_{{x_{2} \gamma_{23} x_{3} }}^{r} (f_{k} ), \ldots ,S_{{x_{B - 1} \gamma_{(B - 1)B} x_{B} }}^{r} (f_{k} )$ denote the corresponding mutual power spectrum, which can be calculated by Eq. (3).

3 $$S_{{x_{1} \gamma_{12} x_{2} }}^{r} (f_{k} ) = [X_{1}^{r} (f_{k} )\gamma_{12} X_{2}^{r*} (f_{k} )]$$

However, according to Eq. (3), the multiplication of the Fourier coefficient and its complex conjugate is applied in CS. If we take Eq. (3) into Eq. (2), there will be terms like $X_{2}^{r *} (f_{k} )X_{2}^{r} (f_{k} ),X_{3}^{r *} (f_{k} )X_{3}^{r} (f_{k} ), \ldots.$ These terms can be merged together resulting in possibly loss of some important information.

Being the spread of Fourier transform, discrete cosine transform (DCT) has the property of energy aggregation (Huang et al. 2014). Its coefficients are sensitive to the change of energy. As a result, the DCS fusion algorithm is proposed based on replacing Fourier transform in earlier CS by DCT, so that the high sensitivity of the DCT coefficients can be employed to improve feature performance. The signals are firstly divided into *K* parts and each part has *M* points. The DCT coefficients are defined by Eq. (4) (Yang et al. 2014).

4 $$\begin{aligned} X_{i}^{j} (0) & = \frac{1}{\sqrt L }\sum\limits_{c = 0}^{L - 1} {x_{i}^{j} (c)} \\ X_{i}^{j} (f_{k} ) & = \sqrt {\frac{2}{L}} \sum\limits_{c = 0}^{L - 1} {x_{i}^{j} (c)\cos \frac{{(2L + 1)f_{k} \pi }}{2L}} \\ \end{aligned}$$

where $x_{i}^{j} (c)$ denotes the *j*th part of the *i*th signal. L is the data length. As a result, in the proposed DCS fusion algorithm, Eq. (1) is redefined as:

5 $$S_{CS} (f_{k} ) = \left( {\sum\limits_{r = 1}^{{n_{s} }} {X_{1}^{r} (f_{k} )\gamma_{12}^{2} X_{2}^{r} (f_{k} )\gamma_{23}^{2} X_{3}^{r} (f_{k} ) \cdots X_{B - 1}^{r} (f_{k} )\gamma_{(B - 1)B}^{2} X_{B}^{r} (f_{k} )} } \right)^{1/B} /n_{s}$$

Compared with Eq. (1), DCS fusion algorithm applies DCT instead of Fourier transform. All coefficients are real numbers. Consequently, Eq. (5) is able to combine Eq. (2) with Eq. (3) to avoid information losing caused by multiplication of Fourier coefficient and its complex conjugate. Furthermore, high sensitivity of DCT coefficients is also inherited. Theoretically, feature performance may be effectively improved.

### Extraction of the DCS composite spectrum entropy

As an effective spectral analysis parameter, spectrum entropy is able to generally describe the performance degradation trend (Liu et al. 2014). Therefore, the DCS composite spectrum entropy (DCSE) is extracted as the feature for later fault prediction. Three-channel vibration signals are taken for research in this paper. Procedures are detailed in the following

1. Divide each signal into *N* parts. Conduct DCT on each part and achieve $X_{i}^{j} ,i = 1,2,3,j = 1, \ldots ,N$.
2. Calculate the correlation coefficient between the *i*th component and the (*i* + 1)th component in the frequency $f_{k}$:
  6 $$\gamma_{i(i + 1)}^{2} (f_{k} ) = \left| {\sum\limits_{j = 1}^{N} {S_{{x_{i} x_{i + 1} }}^{j} (f_{k} )} } \right|^{2} /\sum\limits_{j = 1}^{N} {S_{{x_{i} x_{i} }}^{j} (f_{k} )} \sum\limits_{j = 1}^{N} {S_{{x_{i + 1} x_{i} }}^{j} (f_{k} )}$$
  where, $S_{{x_{i} x_{i + 1} }}^{j} (f_{k} )$ is defined as:
  7 $$S_{{x_{i} x_{i + 1} }}^{j} (f_{k} ) = X_{i}^{j} (f_{k} )X_{{i{ + }1}}^{j} (f_{k} )$$
  where, $X_{i}^{j} (f_{k} )$ denotes the *j*th part’s DCT coefficient in the *i*th signal.
3. Calculate the composite spectrum $S_{CCS} (f_{k} )$:
  8 $$S_{CS} (f_{k} ) = \left( {\sum\limits_{j = 1}^{N} {X_{1}^{j} (f_{k} )\gamma_{12}^{2} X_{2}^{j} (f_{k} )\gamma_{23}^{2} X_{3}^{j} (f_{k} )} } \right)^{1/3} /N$$
4. Calculate the DCSE:
  9 $$\left\{ {\begin{array}{*{20}l} {{\text{DCSE}} = - \left( {\sum\limits_{k = 1}^{K} {p_{i} \ln p_{i} } } \right)/\ln K} \hfill \\ {p_{k} = S_{CS} (f_{k} )/\sum\limits_{k = 1}^{K} {S_{CS} (f_{k} )} } \hfill \\ {\sum\limits_{k = 1}^{K} {p_{k} } = 1} \hfill \\ \end{array} } \right.$$
  where, *K* means the number of frequency bands. Based on fusion of various signals, DCSE is sensitive to the energy changing. When the fault degradation degree is light, the energy distributes in frequency bands in balance and the DCSE will be higher. When the degree is heavy, the energy mainly centered on unique feature frequency bands and the DCSE will be lower.

## Fault predicting based on INW–ESN

Since neurons in the reservoir of ESN are randomly and sparsely connected, this character may lead to the poor guidance ability. It may even affect the generalization and prognostic accuracy (Koryakin et al. 2012). Small world networks possess shorter feature route length, so they perform like the random networks. Furthermore, the polymerization coefficient is higher, small world networks also operate like the regular networks (Zippo et al. 2013; Quan and Zhu 2010). If the small world networks are applied in the reservoir, the generalization may be possibly improved. However, in the NW small world model, the connection weights between nodes are only 0 or 1. Elements in the neighboring matrix are also the inherent 0 and 1, which belongs to the determinacy connection. Limited by this kind of connection, the updating ability of the structure can hardly meet the predicting requirements of the nonlinear and time-varying series. To solve the problem, the improved NW small world network–ESN (INW–ESN) is proposed.

First of all, the NW model is used to make modification of the reservoir structures for improving the generalization ability. The state updating function of INW–ESN is demonstrated in Eq. (10).

10 $$x(k + 1) = f(W_{in} u(k + 1) + Wx(k) + W_{back} y(k))$$

where, $f( \cdot )$ denotes the driving function. *x*(*k*) and *y*(*k*) respectively represent for the state vector and the output vector in the time of *k* and *u*(*k* + 1) is the input vector in the time of *k* + 1. *W*_in_, *W* and *W*_back_ respectively denote the input connection weights matrix, the NW reservoir inner connection weights matrix and the feedback connection weights matrix. The output of is shown in Eq. (11).

11 $$y(k + 1) = f\left( {W_{out} (u(k + 1),\quad x(k + 1),y(k))} \right)$$

where, *W*_out_ is the output weights matrix.

Furthermore, the connecting weight *p* between inner nodes in INW–ESN is defined to make the reservoir update itself topology structure dynamically for better prediction performance. It has been proved that *p* has relations to the distance between neighboring nodes. However, relying solely on the distance to decide *p* may lead to the reduction of the randomness of inner nodes connections. Based on the generally consideration of both distance and the randomness, *p* in IWN–ESN model is defined as:

12 $$p = e^{ - \lambda d} + (1 - e^{ - \lambda d} ) \times rand(0,1)$$

where, the range of *p* is [0,1]. *d* is the Euclidean distance between nodes. $\lambda$ is the adjustment parameter. Based on Eq. (12), *p* is decided by both the distance factor $e^{ - \lambda d}$ and the random factor $rand(0,1)$. When the distance is short, $e^{ - \lambda d}$ is near to 1 and $(1 - e^{ - \lambda d} )$ tends to be 0. Therefore, in this situation, *p* is mainly decided by the Euclidean distance *d*. When the distance is long, $e^{ - \lambda d}$ is near to 0 and $(1 - e^{ - \lambda d} )$ tends to be 1. Therefore, in this situation, *p* is mainly decided by the random factor $rand(0,1)$.

**Fig. 1:**
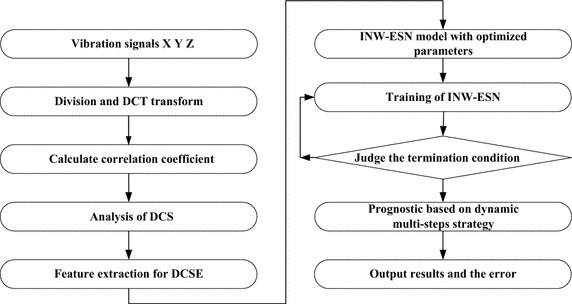
Flowchart of the INW–ESN prognostic

Procedures of the prognostic based upon INW–ESN are detailed in the following. The flowchart is shown in Fig. 1.

- *Step 1* Fuse the three-channel vibration signals by the proposed DCS fusion algorithm and extract the DCSE as the feature (Specific algorithm is indicated in “Extraction of the DCS composite spectrum entropy” section).
- *Step 2* Select the training section and the predicting section from DCSE series and carry out INW–ESN training.
- *Step 2.1* Make optimization of the reservoir scale *EN* and the inner connection weights matrix spectral radius *ER* by the fruit fly optimization algorithm (FOA) (Pan 2012). Achieve the optimal parameters of the INW–ESN.
- *Step 2.2* Conduct the training of INW–ESN according to Eqs. (10), (11).
- *Step 3* Make prediction by the trained INW–ESN based upon the dynamic multi-steps strategy.
- *Step 3.1* After one predicting step, select part of the output to be the predicting results of this step of prediction and update the input vector for the prediction in next step.
- *Step 3.2* Decide whether it meets the termination condition. If it is, combine prediction results of all steps to be the final result. Otherwise, return to the Step 3.1 and go on to the next step of prediction.
- *Step 4* Make analysis and comparison of the predicting result and the real DCSE series. Statistics the errors.

The improvement of the node connection in the proposed INW–ESN ensures that the reservoir is able to update the topology structure dynamically according to various input series. This character not only keeps the sparsity of reservoir neurons, but also reduces the blindness of random connection. Therefore, the predicting accuracy and the network generalization ability are effectively improved in the proposed INW–ESN.

## Experimental validation

### Experimental rig

**Fig. 2:**
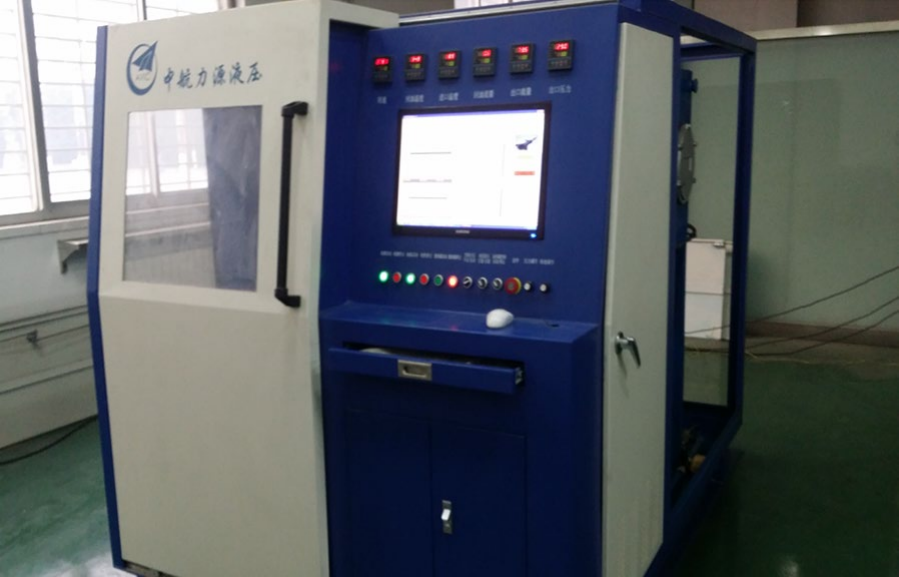
Test-bed for hydraulic pump degradation experiment

**Fig. 3:**
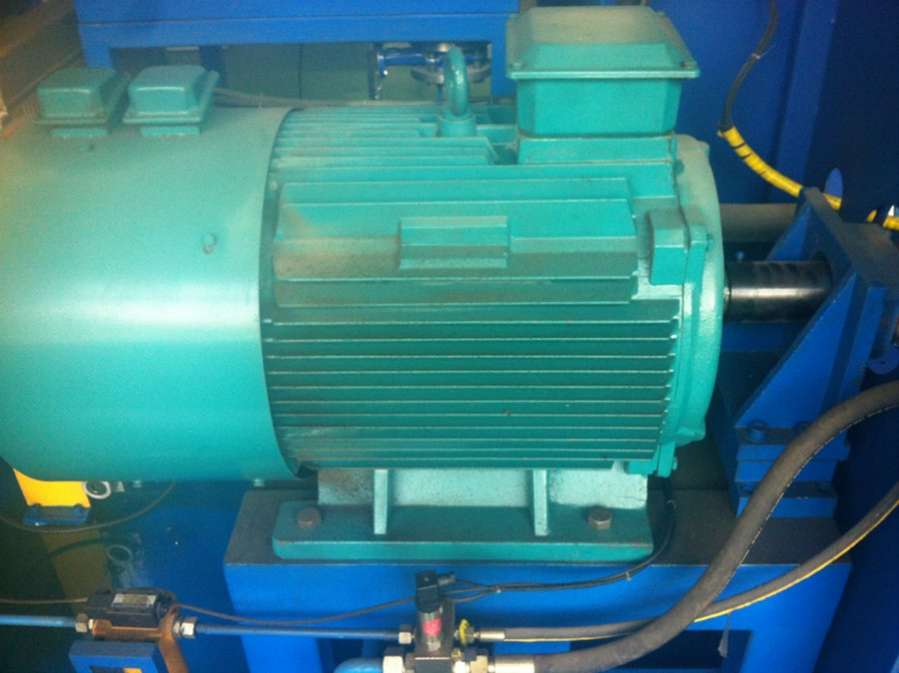
The driving system

**Fig. 4:**
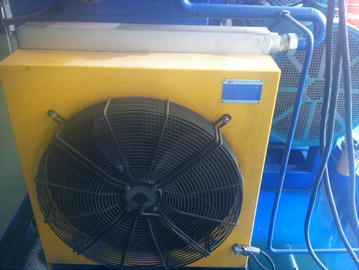
The radiator in the cooling system

In order to achieve the whole lifetime data, the experiment was carried out on the hydraulic pump test-bed, which is shown in Fig. 2. It consists of the driving system, the pressure adjustment system, the control system, the sampling and display system and the cooling system. In the driving system, which is shown in Fig. 3, the driving dynamo was YPT-280M-2. It had the settled speed of 0–3000 rp min^−1^. There are also the components of transducer, coupling and fixture fringe. In the pressure adjustment system, the KBPST8AA/HCG24K4V electromagnetic proportion overflow valve are used to control the outlet pressure of the pump. The control system is embedded in the IPC-610-L using Labview language. The parameters in the control interface contains the outlet pressure, the real speed, the outlet flow, the return oil flow, the inlet temperature, the return oil temperature and the volumetric efficiency. Additionally, there are also adjusting bars of the speed and the pressure, the system start and stop control buttons, the radiator and the calorifier control buttons. In the sampling and display system, there are two turbine flow-meters for monitoring the return flow and the outlet flow, which types are DN6 0.5–16 lpm and DN15 17–120 lpm. Two temperature sensors, WZP-269 and WZP-270, are used to measuring the temperature in the oil box and in the pump inlet. The MPM480 [0–60 MPa] E22B1C1G pressure sensor is applied in the monitoring of outlet pressure. Three vibration sensors (603C01) are used to probe vibrations of the pump. All these signals are sampled by the PCI-6221 data acquisition card. In the cooling system, the type of the radiator is LU-904MF00V501, which is shown in Fig. 4.

**Fig. 5:**
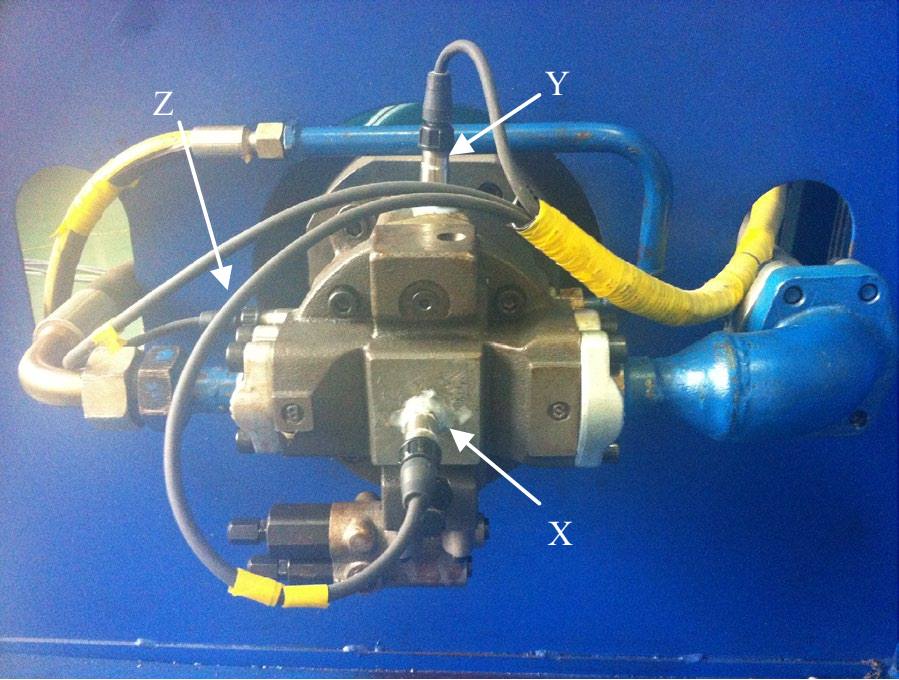
Installation of the vibration sensors

The type of the hydraulic pump tested was L10VSO28DFR. Its rated speed was 2200 r min^−1^ and the working pressure was 26Mpa. The driving dynamo was YPT-280M-2, which had the settled speed of 0–3000 r min^−1^. Since the degradation period of hydraulic pump is so long, in order to improve efficiency, the settled pressure in experiment was 28.3 Mpa and the speed was 2780 r min^−1^. Vibration sensors were respectively installed on X, Y and Z directions of the pump end cap, which is shown in Fig. 5. The three-channel signals were sampled and stored by the cDAQ-9171 system of NI Corporation. The sampling frequency was 5200 Hz and the sampling time was 10 s. The interval time was 23 min.

**Fig. 6:**
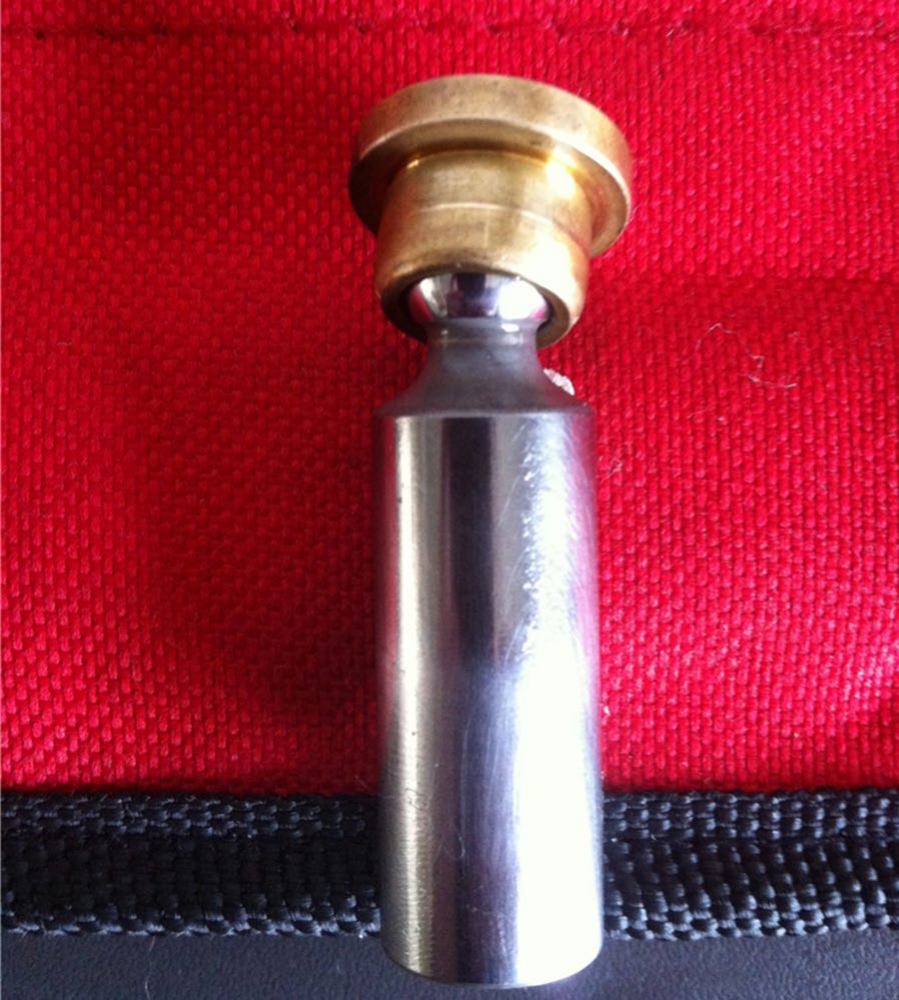
The failure of loose slipper

The volumetric efficiency (VE) is employed as the hydraulic pump performance index in the experimental system. If VE is lower than 80 %, the pump reaches the failure state. In other words, the degradation process is over. In this experiment, when the operating time was 37214 min, VE was lower than 80 %. The pump was affirmed failed by the control system and the experimental operating was shut down automatically. After the experiment, the tested pump was disassembled. The failure mode was confirmed the loose slipper, which is shown in Fig. 6.

The loose slipper shown in Fig. 6 is the failure most easily to occur in the pump degradation. Influenced by the stress during long time operating, the abrasion between the slipper hat and the piston bulb becomes more and more severe. As a result, the distance between the slipper hat and the piston bulb keeps increasing. Additionally, the metal particles caused by abrasion in the pump embed into the distance. When the distance is wide enough, the loose slipper occurs. In this failure condition, the space between the slipper hat and the piston bulb is filled with hydraulic oil. During the movement of the piston, there may be the relative motion between the two parts leading to the strikes on swash plate. If the loose slipper is severe, the piston may be sticking and even the piston bulb happens to break, resulting in catastrophic accident of the hydraulic system. Therefore, it is meaningful to apply the proposed method for the prediction for loose slipper degradation.

### Results and analysis

**Fig. 7:**
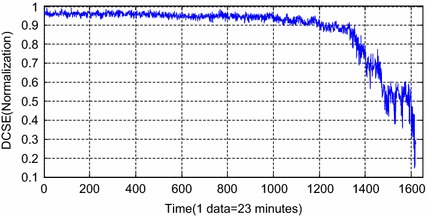
The changing of DCSE after normalization

**Fig. 8:**
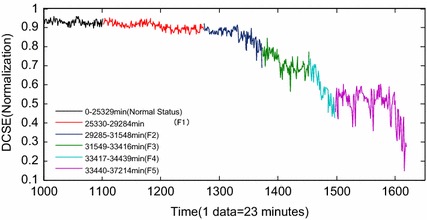
The division of the whole process of DCSE

By the processing of the restored whole lifetime data of the three-channel vibration signals of the hydraulic pump, 1619 groups of samples were achieved. DCSE of each sample is obtained by DCS fusion of the three-channel data. After normalization, the achieved DCSE are shown in Fig. 7. Considering the failure mechanism and the fluctuation tends curve, the whole trend of DCSE in Fig. 7 has been divided into several stages, which is indicated in Fig. 8.

Figure 8 shows that the whole process is divided into 6 parts: during 0–25,329 min (the 1–1101 groups of samples), it is the normal status. In this period, the variety of DCSE is faint and stable; During 25,330–29,284 min (the 1102–1273 groups of samples), the period is the mild wear status (F1). In this stage, the friction pairs in the hydraulic pump had been working for a long time in the accelerated condition. Signs of abrasion occurred because of the continuous friction. Therefore, DCSE tends to decrease; During 29,285–31,548 min (the 1274–1372 groups of samples), it is the slow development status (F2). In this period, the decreasing trend of DCSE become more obvious caused by the aggravation of the continuous friction between the friction pair; during 31,549–33,416 min (the 1373–1453 groups of samples), it is the initial degradation status (F3). There had been signs of some failure mode along with the aggravation of friction. The variety of DCSE is violent and it tends to be momentary steady; During 33,417–34,439 min (the 1454–1497 groups of samples), it is the accelerated degradation status (F4). Since the moment coefficient of the friction pairs became larger, the oil slick became thinner and the fitting clearance became longer. As a result, there were oil leakage and the performance parameters of the pump changed severely. Therefore, DCSE continues decreasing with obvious fluctuation; during 34,440–37,214 min (the 1498–1619 groups of samples), it is the loose slipper status (F5). The metallic particles in the hydraulic fluid had entered into the fitting clearance between the piston cap and the plunger head, which greatly aggravated the friction of the slipper pair. The hydraulic pump performance in this stage can hardly meet the engineering requirements. Therefore, DCSE in this period appears with severe fluctuations.

**Fig. 9:**
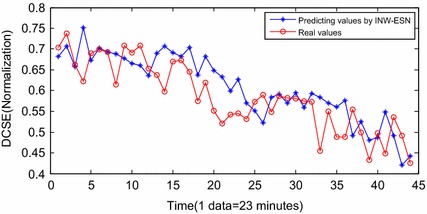
Predicting results by INW–ESN (dynamic)

**Fig. 10:**
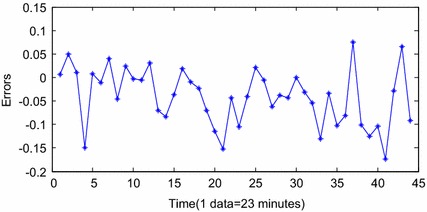
The errors of INW–ESN (dynamic)

Above all, the extracted DCSE is able to reveal the inner relations of the performance degradation of the hydraulic pump. Meanwhile, it is sensitive to the variety of the failure. Therefore, it is reasonable to choose the DCSE to be the whole lifetime evaluation efficient of the hydraulic pump. Based on the extracted DCSE, the prognostic is conducted according to the proposed method in “Fault predicting based on INW–ESN” section. The stage of F3 is selected as the training section of INW–ESN. After optimization by FOA, the optimal parameters are *EN* = 30 and *ER* = 0.5. Since the hydraulic pump performance in stage F5 is confirmed to be invalid, the end time of stage F4 is considered as the occurrence time of the failure. Therefore, stage F4 is selected as the predicting section and the starting point is the 1454th sample. The threshold is settled according to the 1619th sample, which is considered as the occurrence time of failure. The termination condition of failure is DCSE = 0.4251. On the basement of dynamic multi-steps predicting strategy, the operation of INW–ESN reaches the threshold after 43 steps. The results are shown in Fig. 9 and the errors between the predicting data and the real data are shown in Fig. 10.

**Fig. 11:**
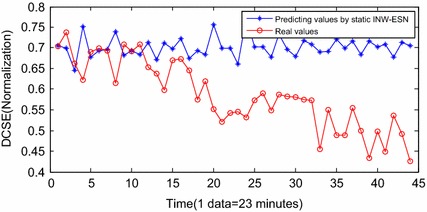
Predicting results by INW–ESN (static)

**Fig. 12:**
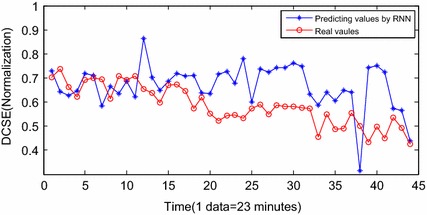
Predicting results by RNN

**Fig. 13:**
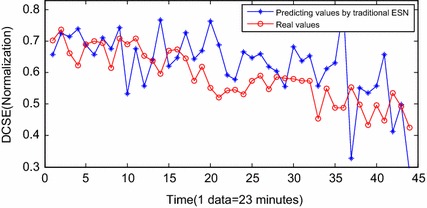
Predicting results by the traditional ESN

**Fig. 14:**
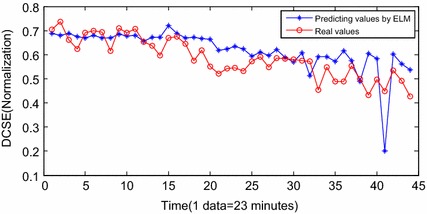
Predicting results by ELM

Figure 9 shows the results of INW–ESN prognostic. Since the reservoir of ESN is improved by the INW model, the elements of the neighboring connection matrix are redefined by the Euclidean distance and the random factors. The reservoir is able to update the topology structure dynamically according to various input series. Therefore, the generalization ability and the prognostic accuracy of INW–ESN are improved. Consequently, the predicting series by the proposed INW–ESN are better fitting the real data and the errors are relatively small. The predicting algorithm reaches the threshold in the 43th sample (for the 1496th sample in Fig. 8) and the failure is confirmed to be occurred. The error of the remaining useful life (RUL) prediction is one data point, which is 23 min. For further indication of the advantages of the proposed method, based on the same training data and prognostic data, the INW–ESN static algorithm, the RNN dynamic algorithm, the traditional ESN dynamic algorithm and the ELM dynamic algorithm are applied for comparison. Results are shown in Figs. 11, 12, 13, 14.

Figure 11 shows the prognostic results by the INW–ESN with the static strategy. The input vector is not updated by the predicting results in each step. The errors between the predicting data and the real data are obvious. The effect is severely worse than those with the dynamic strategy, and the RUL can hardly be predicted based on the current DCSE data. Figure 12 shows the results by the RNN. It can only roughly fit the trend of the stage of F5. Influenced by the back propagation algorithm, RNN is easy to be trapped in local minimums and the convergence rate is also low. Furthermore, there has been large deviation from the actual. In the 38th sample (for the 1491th sample in Fig. 8), the algorithm reaches the threshold. The error of RUL is 6 data points, which is 138 min. Figure 13 shows the results of the traditional ESN. Compared with RNN, the ESN applies the reservoir as the inner network, so that the prognostic effect is improved. However, limited by the sparsely connecting of inner neurons in the reservoir, parts of the predicting results still deviate a little far from the real data. In the 37th sample (for the 1490th sample in Fig. 8), the ESN algorithm meets the termination condition. The RUL predicting error is 7 data points, which is 161 min. Figure 14 shows the results of ELM prognostic. By the abandon of the iteration strategy of gradient descent, ELM has characteristics as simple parameters, fast learning and better searching ability. As a result, the predicting curve is closer to the real one. However, influenced by the input weights vector and the random selection of hidden nodes’ weights, the ELM algorithm reaches the termination condition in the 41th sample (for the 1494th sample in Fig. 8). The RUL predicting error is 3 data points, which is 69 min.

Based on the above comparison, the proposed method shows obvious advantages in prognostic. For further quantitative evaluation, the mean absolute percentage error (MAPE) and the root mean square percentage error (RMSPE) are selected as the evaluation indexes. MAPE and RMSPE of the proposed method and the above 4 method are achieved and the results are shown in Table 1.

**Table 1:** MAPE and RMSPE of various algorithms

Algorithms	MAPE (%)	RMSPE (%)	Error of RUL prediction
INW–ESN (dynamica)	9.32	7.27	23 min
INW–ESN (static)	17.14	14.64	Failed
RNN (dynamica)	16.38	13.44	138 min
ESN (dynamica)	14.60	10.87	161 min
ELM (static)	13.91	9.48	69 min

Table 1 shows that, because of the updating of the input vector, the algorithms with the dynamic strategy perform better than those with the static strategy. Since the reservoir is modified and the element in the neighboring matrix is redefined, the prognostic performance of INW–ESN has been effectively improved. Therefore, the error for RUL prediction is only 23 min, and the MAPE and RMSPE are also the lowest compared with other four algorithms.

**Fig. 15:**
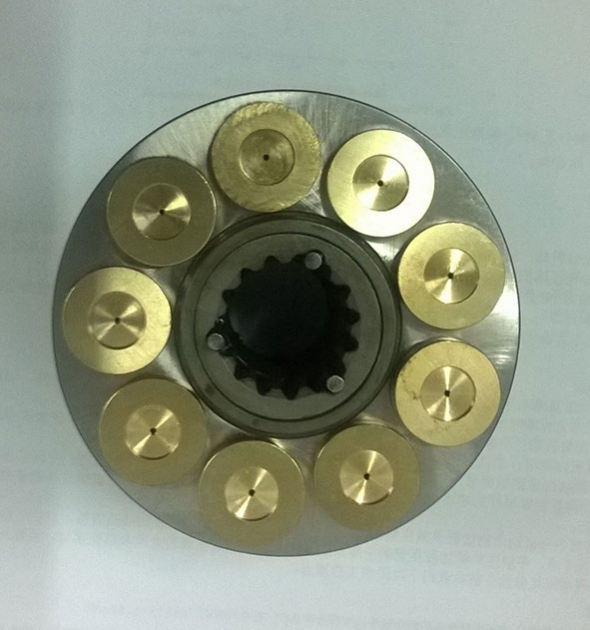
The failure of slipper abrasion

To further verify the efficiency, other three degradation experiment conducted previously are applied. The type of the pump and the parameters setting were the same. The whole lifetime of these three pump in the experiments were 38,801, 40,365 and 2,8014 min. The correspondent final failure modes were loose slipper, loose slipper and slipper abrasion (Fig. 15). The proposed method, together with the above four methods, are employed for making prediction by processing the experimental data. Results are shown in Table 2.

**Table 2:** Results of the application in other degradation experiments

Algorithms	Loose slipper failure			Loose slipper failure			Slipper abrasion failure		
	MAPE (%)	RMSPE (%)	Error	MAPE (%)	RMSPE (%)	Error	MAPE (%)	RMSPE (%)	Error
INW–ESN (dynamica)	10.17	7.89	23 min	13.11	9.03	46 min	6.25	4.69	0 min
INW–ESN (static)	20.43	16.97	Failed	25.92	19.56	Failed	14.35	10.08	Failed
RNN (dynamica)	18.29	15.11	161 min	21.05	16.47	184 min	11.17	8.83	69 min
ESN (dynamica)	16.08	12.14	207 min	19.38	13.92	253 min	8.41	6.05	92 min
ELM (static)	14.87	10.93	92 min	16.77	12.01	115 min	7.86	5.33	46 min

In Table 2, compared with other algorithms, the proposed method is also able to achieve the best prognostic performance. The MAPE and the RMSPE are the highest and the error is the lowest. Furthermore, the prediction accuracy of the proposed INW–ESN for the slipper abrasion failure is obviously higher than that for the loose slipper. The reason can be explained that the slipper abrasion failure mechanism is much simpler and the degradation time is less. Combined with Table 1, results shows that the proposed method is feasible in the prediction for various failure modes.

From the above analysis, we conclude that the proposed DCS and INW–ESN algorithm performs much better in prognostic. The feature information is effectively extracted by the fusion of multi-channel vibration signals based on DCS. Furthermore, the reservoir is better improved in the INW–ESN for making prognostic accurately and effectively. However, there are also some problems that should be the focus of further study: (1) only one feature was extracted in this paper, and this may lead to the loss of some important information. To solve this problem, other typical features could also be extracted and the information fusion algorithm will be introduced to make fusion of all the extracted features so as to better obtain fault information and to improve the feature performance; (2) The research in this paper represents the initial application of the presented method to make fault prediction. And the research on combination prognostic method to further improve the predicting effects was not involved. To make fault prediction of the hydraulic pump more accurately and meaningful, the research that addresses the problems mentioned above will be reported in our future work.

## Conclusions

 A novel method for fault prediction of hydraulic pump based on DCS and INW–ESN is proposed, which is verified by the whole lifetime data sampled from the hydraulic pump degradation experiment. Conclusions can be drawn as follows:

1. The DCS algorithm is presented and the multi-channel vibration signals are fused. Meanwhile, the DCS spectrum entropy with high sensitivity is extracted to be the degradation feature.
2. The INW–ESN is proposed for prognostic for hydraulic pump. The reservoir is modified and the disadvantages caused by the sparsely connection are solved. The generalization ability and predicting accuracy of the network have been effectively improved.
3. Results of the application in the hydraulic pump degradation experiment show that the proposed method is feasible and the predicting accuracy is satisfactory, which is meaningful for CBM.
